# Antiprotozoal Activities of *Millettia richardiana* (Fabaceae) from Madagascar

**DOI:** 10.3390/molecules19044200

**Published:** 2014-04-03

**Authors:** Manitriniaina Rajemiarimiraho, Jean-Théophile Banzouzi, Marie-Laure Nicolau-Travers, Suzanne Ramos, Zakaria Cheikh-Ali, Christian Bories, Olga L. Rakotonandrasana, Stéphane Rakotonandrasana, Philippe Antoine Andrianary, Françoise Benoit-Vical

**Affiliations:** 1 Centre National d’Application de la Recherche Pharmaceutique (CNARP), BP 702 Antananarivo 101, Madagascar, France; E-Mails: manitrarajemi@yahoo.fr (M.R.); stephanandrasana@yahoo.fr (S.R.); 2 Centre d’Etude et de Recherche Médecins d’Afrique (CERMA), 43, rue des Glycines, 91600 Savigny sur Orge, France; E-Mail: olala200052@yahoo.fr; 3 Ecole Supérieure Polytechnique d’Antananarivo (ESPA), Université d'Antananarivo, BP 1500, Antananarivo 101, Madagascar; E-Mail: espa.tana@gmail.com; 4 Institut de Chimie des Substances Naturelles (ICSN-CNRS), Bâtiment 27, 1 Avenue de la Terrasse, 91198 Gif-sur-Yvette Cedex, France; E-Mail: suzanne.ramos@icsn.cnrs-gif.fr; 5 CNRS/LCC (Laboratoire de Chimie de Coordination) UPR8241; 205, route de Narbonne, F-31077 Toulouse, France; 6 Université de Toulouse III; UPS, LCC; 118, route de Narbonne, F-31077 Toulouse, France; E-Mail: marielaure9697@hotmail.fr; 7 Service de Parasitologie-Mycologie, Centre Hospitalier Universitaire de Rangueil, Université de Toulouse et Faculté de Médecine de Rangueil, Université de Toulouse III; UPS, TSA 50032, 31059 Toulouse cedex 9, France; 8 Laboratoire de Pharmacognosie et de Chimiothérapie Antiparasitaire, CNRS UMR 8076 BioCIS, Faculté de Pharmacie, 5 rue J.-B. Clément, Université Paris-Sud 11, 92296 Châtenay-Malabry, France; E-Mails: zakaria.cheikh-ali@u-psud.fr (Z.C.-A.); christian.bories@cep.u-psud.fr (C.B.)

**Keywords:** Millettia richardiana, traditional medicine, Madagascar, Plasmodium, Trypanosoma, Leishmania, Candida

## Abstract

With at least 60% of the *Millettia* species (Fabaceae) being in medicinal use, we found it relevant to assess the potential antiprotozoal and antifungal activities of *Millettia richardiana.* Water and methanol crude extracts of the stem barks from *M. richardiana* and the six fractions resulting from the fractionation of the methanol extract were tested. The dichloromethane extracted fraction showed the best *in vitro* antiprotozoal activities (IC_50_ = 5.8 μg/mL against *Plasmodium falciparum*, 11.8 μg/mL against *Leishmania donovani* and 12.8 μg/mL against *Trypanosoma brucei brucei*) as well as low cytotoxicity on several cell lines. The phytochemical analysis showed this selected fraction to be rich in terpenoids and alkaloids, which could explain its antiparasitic activity. A phytochemical study revealed the presence of lonchocarpenin, betulinic acid, β-amyrin, lupeol, palmitic acid, linoleic acid and stearic acid, among which betulinic acid and lupeol could be the compounds responsible of these antiprotozoal activities. By contrast, neither the crude extracts nor the fractions showed antifungal activity against *Candida*. These results confirm the importance of the genus *Millettia* in Malagasy ethnomedicine, its potential use in antiparasitic therapy, and the interest of developing a sustainable exploitation of this plant. Moreover, both molecules betulinic acid and lupeol appeared as very relevant molecules for their antiprotozoal properties.

## 1. Introduction

In a previous paper, we listed all the medicinal or toxic species of *Millettia* from subtropical Africa and Madagascar and showed that about 60% of the species in the genus *Millettia* are known for their medicinal properties [1]. Eight *Millettia* *species* are present in Madagascar: *M. aurea, M. capuronii*, *M. hitsika, M. lenneoides*, *M. nathaliae*, *M. orientalis*, *M. richardiana*, and *M. taolanaroensis*. The Malagasy *Millettia* most known to be medicinal or toxic are *M. lenneoides* and *M. taolanaroensis* (insecticidal properties, fishing poison). Three other species, known under the name *Millettia* in the traditional Malagasy pharmacopoeia*,* but now assigned to the *Pongamiopsis* or *Mundulea* genera, are also used*: M. pervilleana* and *M.* *chapelieri* (insecticidal properties and fishing poisons) and *M. amygdalina* (syphilis). Several researchers, and mostly the team of Rasoanaivo, have begun to validate the pharmacological potential of the Malagasy *Millettia* species in the laboratory, essentially for their anticancer activity [2,3,4]. All these eight *Millettia* are endemic species in Madagascar and most are threatened or vulnerable according to IUCN standards [5]. *M. lenneoides* and *M. richardiana* are the least endangered species. Nothing has been published on these plants, but as the medicinal potential of *M. lenneoides* is already known, we choose to assess the biological properties of *M. richardiana*. 

The present study was thus undertaken to assay the antiparasitic potential of *Millettia richardiana* (Baill.) Du Puy & Labat (Synonyms: *M. baronii* Drake, *Mundulea hysterantha* Baker, *Mundulea richardiana* Baill., *Neodunnia atrocyanea* R. Vig.), one of the still plentiful species, which had not yet been studied despite its broad distribution. *M. richardiana* is a small tree (up to 10 m in height) or a shrub of the dry deciduous forests growing on limestone, sand or on dunes in the west of Madagascar, below 300 m altitude, from Toliary to Antsiranana. The young stalks are covered with brown hairs. The leaves are alternate, compound with seven pairs of leaflets; the rachis and veins are also covered with gold-coloured brown hairs. Inflorescences in the axillary position are pseudo-racemes of 2-4 flowers only. The fruits are obovate flattened pods, ending in a bent beak, 9 cm long, covered with yellowish soft hairs. According to the regions, the vernacular names differ: Hararandrato, Hazovola, Lovanjafy, Marary, Mararybotry, Mararytoloha, Manaribôraka, Taintsindambo, Tankiandambo, Taitsindambo, Tsimahamasabary. 

We report here the results of the antiprotozoal (against *Plasmodium falciparum*, *Trypanosoma brucei*, *Leishmania donovani*) and fungicidal (against *Candida albicans, Candida glabrata* and *Candida parapsilosis*) assays of two crude extracts and six fractions from the stem bark of *M. richardiana*. Their cytotoxicity was also evaluated on KB, Vero and MRC5 cells. These *M. richardiana* biological data led to bio-phytochemical correlation between active principles and pharmacological results.

## 2. Results and Discussion

Malaria, African trypanosomiasis and leishmaniasis are a group of tropical diseases that are especially endemic in low-income populations in developing regions of Africa, Asia and South America. Together, these three parasitic diseases cause up to an estimated 2 million deaths annually [6]. Otherwise, Candidemia are opportunistic fungal infections causing alarming problems with serious infections, especially in immunologically compromised people [7]. Natural products have played a key role throughout the history of drug discovery as a source of both novel compounds. Many plants are daily used in tropical and sub-tropical areas to treat infectious diseases. In this context, as at least 60% of the *Milletia species* being known for their medicinal properties, we have thought relevant to assess the potential antiprotozoal and antifungal activities of *M. richardiana,* even if we found no written record of its ethnobotanical use. The two crude aqueous (traditional) and methanol (MRE1) extracts from *M. richardiana* showed extraction yields of 10.2% and 8.4%, respectively whereas the 6 fractions from MRE1 had extraction yields ranging from 4.1% to 43.0% (Table 1). 

**Table 1:** Extraction yields and chemical classes of compounds found in the extracts and fractions from *M. richardiana.*

Extracts and Fractions ^a^	Solvent	Weight (g)	Yield (%)	Chemical classes ^b^
Traditional aqueous extract	Water	15.2	10.2	/
MRE1	MeOH	84.0	8.4	/
MRE2	DCM	21.8	27.2	TE, AL
MRE3	AcOEt	3.3	4.1	TE, AL, SA
MRE4	BuOH	20.0	25.6	SA, PH, SU
MRE4A	BuOH	0.5		SA, FL
MRE4B	BuOH	14.2		FL, PA, SU
MRE5	Water	34.4	43.0	TA, FL

^a^ MRE1: Crude MeOH Extract; MRE2: DCM Fraction; MRE3: AcOEt Fraction; MRE4: BuOH Fraction; MRE5: Aqueous Fraction. Fractions MRE2 to MRE5 were extracted from MRE1. Fractions MRE4A and MRE4B were extracted from MRE4. ^b^ AL: Alkaloids; FL: Flavonoids; TE: Terpenoids; SA: Saponins; TA: Tannins; PH: Phenolics; PA: Phenolic acids; SU: Sugars.

The chemical classes present in *M. richardiana* were identified through the TLC screening and the most represented were saponins, flavonoids, terpenoids, alkaloids, sugars, tannins, phenolics and phenolic acids (Table 1). 

Results concerning the antiprotozoal, antifungal and cytotoxicity assays for the extracts and fractions of *M. richardiana* are reported in Table 2. The aqueous crude extract, following the traditional preparation, showed neither activity whatever the pathogen tested nor any cytotoxicity. No activity was also found with the other crude extract in methanol (MRE1). Both types of extraction did not seem to extract the active compounds. Furthermore, two other fractions MRE3 (ethyl acetate) and MRE4A (butanol), containing terpenoids, alkaloids, saponosids, and flavonoids showed weak activity against *Trypanosoma* and *Leishmania*. However MRE4A was the most cytotoxic of the extracts and fractions (61% of cell growth inhibition for KB line). It should be noted that all *M. richardiana* extracts and fractions tested had IC_50_ higher than 10 μg/mL on *Candida sp.*, showing no antifungal activity at pharmacological level, by comparison with the control drug (5-fluorocytosine) active at 0.75 µg/mL. 

On the contrary, the DCM fraction MRE2 extracted from MRE1, shows more promising IC_50_ values against the protozoan parasites tested compared with the control drugs (IC_50_: 5.8 μg/mL against *P. falciparum*, 12.5 μg/mL against *T. brucei brucei* and 11.8 μg/mL against *L. donovani*). The *P. falciparum* data are particularly interesting since IC50 values around 5 μg/mL are considered as good in the laboratory efficacy score for *in vitro* antiplasmodial activity of crude extracts and correspond to the concentration value warranting bioassay-guided fractionation [8]. Moreover, the trypanocidal activity of the MRE2 was comparable to the control drug (IC_50_ = 12.5 µg/mL for MRE2 *versus* 7.4 µg/mL for pentamidine) and leishmanicidal activity was only less than 10 times lower than that of the control drugs (11.8 µg/mL *versus* 1.4 µg/mL for pentamidine and 1.2 µg/mL for miltefosine). These values are also quite promising since they were obtained from crude extracts (grouping many compounds and so diluting the pharmacological activity) and were compared with control drugs corresponding to pure molecules. The good results on all these three tropical parasites thus support the pharmacological potential of this fraction. Furthermore, MRE2 presented a low cytotoxicity with percentages of inhibition ranging from 15% to 22% at 10 µg/mL. DCM extraction is known to give fractions that are the most active in ethnopharmacological studies [9]. The preliminary phytochemical analysis showed this selected fraction to be rich in terpenoids and alkaloids, which could explain its antiparasitic activity. This DCM fraction MRE2 was then chromatographed over a silica gel column eluting successively with CH_2_Cl_2_ and mixtures of CH_2_Cl_2_-CH_3_OH, and yielded seven compounds: lonchocarpenin, betulinic acid, β-amyrin, lupeol, palmitic acid, linoleic acid and stearic acid [10]. β-amyrin, palmitic acid, linoleic acid and stearic acid have no antiprotozoal activity reported. By contrast, lupeol is known to show antiplasmodial (with IC_50_ obtained *in vitro* ranging from 5 to 45 µg/mL) and trypanocidal (IC_50_: from 4 to 19.3 μM) activities [11]. Moreover, betulinic acid exhibited moderate *in vitro* activities against *P. falciparum* (IC_50_: 9.89 μM) [12], against *L. major* (IC_50_: 40 μM) [13] but also against *T. brucei* [14]. Therefore, lupeol and betulinic acid might both explain the antiprotozoal activity of the fraction MRE2.

**Table 2:** Biological activities of extracts and fractions of *M. richardiana.*

	Antiparasitic Activity				Antifungal Activity			Cytotoxicity(% of inhibition at 10^−5^ g/mL)		
Extracts and Fractions ^a^	*P. faciparum FcM29* IC_50_ (µg/mL)	*T. brucei brucei* MAC (µg/mL)	*L. donovani* IC_50_ (µg/mL)		*C. albicans* MIC (µg/mL)	C. glabrata MIC (µg/mL)	*C. parapsilosis* MIC (µg/mL)	KB	Vero	MRC5
Traditional extract	>10	>100	>100		>10	>10	>10	17	0	0
MRE1	>10	50	50.8 ± 2.3		>10	>10	>10	0	0	0
MRE2	5.8 ± 0.9	12.5	11.8 ± 1.8		>10	>10	>10	21	22	15
MRE3	>10	25	47.8 ± 5		>10	>10	>10	0	21	12
MRE4	>10	>100	>100		>10	>10	>10	23	14	20
MRE4A	>10	50	25.8 ± 1.1		>10	>10	>10	61	20	20
MRE4B	>10	>100	>100		>10	>10	>10	0	9	0
MRE5	>10	>100	77.5 ± 3.3		>10	>10	>10	7	3	0
Control Drugs	Chloroquine	Pentamidine isethionate	Pentamidine isethionate	Miltefosine	5-Fluoro-cytosine	5-Fluoro-cytosine	5-Fluoro-cytosine	Taxotere	Taxotere	Taxotere
	230.10^−3 ^± 0.4	7.4 ± 1.3	1.4 ± 0.12	1.2	0.75 ± 0.01	0.75 ± 0.01	0.75 ± 0.01	75	29	90
	(445.10^−3^ µM)	(12.5 µM)	(2.5 µM)	(3.0 µM)	(5.8 µM)	(5.8 µM)	(5.8 µM)	(for 2.5 10^−10^ M)	(for 2.5 10^−10^ M)	(for 2.5 10^−10^ M)

^a ^Traditional Extract: aqueous decoction; MRE1: Crude MeOH Extract; MRE2: DCM Fraction; MRE3: AcOEt Fraction; MRE4: BuOH Fraction; MRE5: Aqueous Fraction.

The *Millettia* genus is already largely known for its antiprotozoal activities, in Africa as well as in Asia. In Africa, *M. griffoniana* had trypanocidal activity due to griffonianone E (IC_50_ = 8.6 µg/mL on *T. brucei brucei* and 6.35 µg/mL on *T. b. rhodesiense*) and antiplasmodial activity due to the presence of griffonianone E, 7-methoxyebenosin and prenylated isoflavonoids, with IC_50_ ranging from 28.3 µg/mL to 36.7 µg/mL [15,16]. *M. usaramensis* showed also antiplasmodial properties (IC_50_ = 21 µg/mL) which its most active compound (IC_50_ = 8.7 µg/mL) against *P. falciparum*, was a chalcone [17]. *M. zechiana* and *M. versicolor* presented moderate antiplasmodial activity (IC_50_ = 16.1 µg/mL and 33 µg/mL, respectively) and low cytotoxicity [18]. Anti-leishmanial properties (IC_50_ = 32 µM for 6,7-dimethoxy-3',4'-methylenedioxyisoflavone on *L. infantum*) and low cytotoxicity (IC_50_ = 43 µM on MRC-5 cells) [19] were also reported for *M. puguensis*. 

In Asia, Nguyen-Pouplin *et al.* [20] demonstrated the antiplasmodial activity of *M. diptera* bark (IC_50_ = 6.2 µg/mL and 5.5 µg/ml for DCM and cyclohexane extracts, respectively) but also a strong cytotoxicity. In Myanmar, from 75 vegetals belonging to 27 families, *M. pendula* had the most potent leishmanicidal activity; Pendulone, an isoflavane extracted from this plant showed a very high leishmanicidal (IC_50_ = 0.07 µg/mL on *L. major*) [21,22].

It should be noted that all the antiprotozoal compounds isolated from these various *Millettia spp* are phenolic compounds belonging to the groups of isoflavans, isoflavonoids, rotenoids and pterocarpans, whereas the fraction MRE2, according to our preliminary analysis, appears to contain little or no flavonoids and other phenolic compounds. The active molecules that are present in the fraction MRE2 seem to belong principally to terpenoids as suggested by the results of the phytochemical analysis.

## 3. Experimental

### 3.1. Plant Material

*M. richardiana* (Baill.) Du Puy & Labat (Fabaceae) was identified and collected by the botanist Stéphane Rakotonandrasana of Centre National d’Application des Recherches Pharmaceutiques (CNARP), in June 2008 at Sahafary (12° 35’ 880S, 049° 26’ 853E), a rural district of Andrafiabe, Diana’s Region, in the ex-Province of Antsiranana. Vouchers specimens were deposited at the CNARP and at the Zoological and Botanical Park of Tsimbazaza (TAN) under n°1342. The stem barks were dried on a cement floor in a well-aerated and dark room over 2 weeks. They were then crushed before being extracted.

### 3.2. Preparation of Plant Extracts

In order to be close to traditional methods of preparation of this medicinal plant, a decoction at 10% was prepared by boiling stem bark powder (150 g) for 10 min in distilled water (1.5 L). The decoction was left to cool for 4 h, filtered through paper and then centrifuged at 3,000 g for 20 min. The supernatant was collected and freeze-dried to yield crude aqueous decoction (traditional extract). In parallel, the stem barks powder (1 kg) was first macerated in methanol (MeOH) at room temperature under shaking (3 × 48 h). The resulting extracts were pooled, filtered through paper and then concentrated at 35–40 °C to give **MRE1** (MeOH Extract). After being solubilised in water, 80 g of MRE1 were fractioned by liquid/liquid extraction successively with dichloromethane (DCM) (Fraction **MRE2**), then with ethyl acetate (AcOEt) (Fraction **MRE3**), and finally with butanol (BuOH) (Fraction **MRE4**). The residual aqueous phase was freeze-dried to give Fraction **MRE5**. Fraction MRE4 was fractioned again by solubilisation in MeOH followed by precipitation in ether (MeOH/ether: 1/4). Several successive precipitations produced **MRE4A** (Precipitate) and the residual liquid phase was freeze-dried to give the fraction **MRE4B** (Lyophilisate). The yields are given in Table 1. MRE2 was chromatographed over a silica gel column (160 g), eluting successively with CH_2_Cl_2_ and mixtures of CH_2_Cl_2_-CH_3_OH (97:3, 95:5 and 90:10), to give 6 fractions, E2A (CH_2_Cl_2_, 3.8 g), E2B (CH_2_Cl_2_, 1.1 g), E2C (CH_2_Cl_2_, 1.6 g), E2DE [CH_2_Cl_2_-CH_3_OH (97:3) 8.5 g], E2F [CH_2_Cl_2_-CH_3_OH (95:5),1.6 g] and E2G [CH_2_Cl_2_-CH_3_OH (90:10), 1.5 g [10].

### 3.3. Chemical Characterisation by TLC Screening

The extracts were screened for the presence of alkaloids, flavonoids, lactones/esters, terpenoids, iridoids, anthraquinones, saponins, tannins, and protein/amino acids on TLC silicagel 60 F254 plates (Merck). Development was carried out with the following solvent system: chloroform/ethyl acetate/methanol/water (17:47:26:10). The positive controls used were: cinchonidine (alkaloids), quercetin (flavonoids), γ-valerolactone (lactones/esters), L-Trp, L-Phe (amino-acids, proteins), lupeol (terpenoids), catalpol (iridoids), 9,10-anthraquinone (anthraquinones), saponarioside C (saponins), ellagic and gallic acids (tannins), glucose (carbohydrates). After development, the plates were sprayed with the appropriate reagents: Dragendorff reagent (alkaloids), Neu reagent (flavonoids), hydroxylamine-ferric chloride (lactones/esters), ninhydrin (protein/amino acids), Liebermann reagent (terpenoids), vanilline-sulphuric acid (iridoids), KOH (anthraquinones), anisaldehyde-sulphuric acid (saponins), ferric chloride (tannins). Reagents were prepared according to Stahl method [23]. Detection was carried out visually in visible light and under UV light (λ = 254 nm or 365 nm). Identification of molecules was then carried out by electrospray mass spectrometry, 1D and 2D NMR methods and GC/MS techniques as detailed in Rajemiarimiraho *et al.* 2013 [10].

### 3.4. Biological Assays

Assays on *Leishmania donovani* and *Trypanosoma brucei brucei* were performed at the Antiparasitic Chemotherapy Department of the Faculty of Pharmacy of Châtenay-Malabry University Paris-Sud 11, France. The experiments on *Plasmodium falciparum* and on *Candida* *spp* were performed at the Parasitology-Mycology Department, Toulouse Hospital University, France. The KB, Vero and MRC5 cells were cultured at the Institut de Chimie des Substances Naturelles (ICSN-CNRS), Gif-sur-Yvette, France.

#### 3.4.1. Antiplasmodial Activity

The extracts and fractions were screened for their antiplasmodial activity on FcM29-Cameroon, a chloroquine-resistant strain of *Plasmodium falciparum* (IC_50_ for chloroquine = 445 nM) as previously reported by Desjardins *et al.* [24] and modified as follows. Extract dilutions were tested 3 to 5 times independently, each dilution in triplicate, in 96-well plates with cultures at a parasitaemia of 1% and a haematocrit of 1%. For each test, the plates of parasite culture were incubated with plant extracts for 48 h and radioactive hypoxanthine was added to the medium 24 h after the beginning of incubation [25]. The control parasite culture (without drug or with 2% DMSO) was referred to as 100% growth. Parasite growth was estimated by [3H]-hypoxanthine incorporation. Concentrations of extracts inhibiting 50% of the parasite growth (IC_50_) were graphically determined as concentration *versus* percent inhibition.

#### 3.4.2. Leishmanicidal Activity

The test was performed as previously described by Mbongo *et al.* [26] and Okpekon *et al.* [27]. Promastigotes of *Leishmania donovani* were grown at 27 °C in a 5% CO_2_ atmosphere in the dark, in M199 medium containing 10% fetal calf serum (FCS) and supplemented with 40mM HEPES, 100mM adenosine, 0.5 mg hemin/L, and 50 mg gentamycin/ml with concentrations of extract . After 1 h at 27 °C under a 5% CO_2_ atmosphere, culture medium containing 1.75 × 10^6^ parasites/mL, from a logarithmic phase culture, was added. After a 72 h incubation period, the viability of parasites was evaluated by the tetrazolium-dye (MTT) colorimetric method. The results are expressed as the concentration inhibiting parasite growth by 50% (IC_50_). Pentamidine isethionate and miltefosine were the reference drugs. These tests were performed three times and each concentration was tested in duplicate (*n* = 6).

#### 3.4.3. Antitrypanosomal Activity

The antiparasitic activity was assessed using the method described in Loiseau *et al.* [28]. Bloodstream forms of *Trypanosoma brucei brucei* were maintained *in vitro* for 48 h in the dark at 37 °C in a 5% CO_2_ atmosphere, in minimum essential medium (Gibco-BRL, Life Technologies, Cergy Pontoise, France) including 25 mM HEPES and Earle’s salts, and supplemented with 2 mM L-glutamine, 1 g of additional glucose per liter, 10 mL of minimum essential medium non-essential amino acids (100×; Gibco-BRL) per liter, 0.2 mM 2-mercaptoethanol, 2 mM sodium pyruvate, 0.1 mM hypoxanthine, 0.016 mM thymidine, 15% heat-inactivated horse serum (Gibco-BRL), and 50 mg gentamycin per milliliter. The minimum active concentration (MAC) was defined as the minimum concentration at which no viable parasite was observed microscopically. Pentamidine was the reference drug. These tests were performed three times and each concentration was tested in duplicate (*n* = 6).

#### 3.4.4. Antifungal Activity

The antifungal activity of the extracts and fractions was evaluated on *Candida albicans* American Type Culture Collection 90028 (ATCC 90028), *C. parapsilopsis* (ATCC 22019) and *C. glabrata* (ATCC 24220). A micro-dilution method adapted from the Clinical and Laboratory Standards Institute (CLSI, formerly NCCLS-M27A) [29] was used. The culture medium used was RPMI 1640 (Sigma, Saint-Quentin Fallavier, France) with 2 mM L-Glutamine and 0.165 M morpholinopropanesulfonic acid (MOPS) buffer (Sigma). Inoculates were prepared by suspending the yeast in a sterile saline solution and adjusting to a final concentration of 2.5 × 10^6^ yeast cells/mL. 100 µL of this suspension was added to each well of 96-well plates and 100 µL of various concentrations of drugs added (with the maximum concentration at 10 µg/mL), and the plates incubated for 48 h at 35 °C. The results were determined using a spectrophotometer (Elx 808, Vetra Microplate Reader, Avantec, Illkirch, France) at a wavelength of 550 nm. The MIC (Minimal Inhibitory Concentration) was determined graphically by plotting concentrations of tested drugs *versus* percentage inhibition. For comparison, the control 5-fluorocytosine was routinely used as a reference in this test.

#### 3.4.5. Cytotoxicity

The cytotoxicity of *M. richardiana* extracts and fractions was tested against KB (human epidermoid carcinoma), Vero (African green monkey kidney) and MRC5 (Normal Human Fetal Lung Fibroblast) cells. These cells were grown in Dulbecco’s modified Eagle’s medium supplemented with 25 mM glucose, 10% (*v/v*) foetal calf serum, 100 UI penicillin, 100 µg/mL streptomycin and 1.5 µg/mL fungizone and kept under 5% CO_2_ at 37 °C. Microplates of 96-wells were seeded with 600 KB cells per well in 200 µL medium. After 24 h, extracts dissolved in DMSO, at a concentration of 10 mg/mL, were added to the cells for 72 h at a final concentration of 1% in a fixed volume of DMSO. Controls received an equal volume of DMSO. After two hours of incubation with the MTS reagent (Promega, Madison, WI, USA), the number of viable cells was determined by measuring the optical density of each well at 490 nm with a spectrophotometer. The positive control used was taxotere (Rhône-Poulenc, Sanofi, Paris, France).

## 4. Conclusions

Despite absence of biological activity for crude extracts, the fractionation of the methanol extract of the stem bark of *M. richardiana* yielded a fraction, active against the parasites *P. falciparum*, *L. donovani* and *T. brucei brucei*. This dichloromethane fraction was particularly promising for its antiprotozoal activities especially against *L. donovani* and *T. brucei brucei* with results close to those obtained with the control drugs and its low cytotoxicity. These findings support the interest in the genus *Millettia* as antiparasitic plants in the traditional pharmacopoeia. Some active compounds such as lupeol and betulinic acid here isolated can explain this pharmacological activity. Further investigations by bio-guided fractionations could permit to identify all the active antiparasitic compounds. *M. richardiana* is currently the object of trials of cultivating with the aim of developing a sustainable exploitation of the plant, without endangering the Malagasy biodiversity.
